# Une étiologie rare de kyste médiastinal

**DOI:** 10.11604/pamj.2014.19.24.3126

**Published:** 2014-09-10

**Authors:** Hicham Fenane, Mohamed Bouchikh, El mehdi Maidi, Damsane Lamboni, Abdellah Achir, Fahd Ouchen, Mbola Oyali, Mohamed Caidi, Said Al Aziz, Abdellatif Benosman

**Affiliations:** 1 Service de chirurgie thoracique hôpital Ibn Sina, Rabat, Maroc

**Keywords:** Etiologie, kyste médiatisnal, goitre ectopique endothoracique, Etiology, mediastinal cyst, ectopic intrathoracic goiter

## Abstract

Le goitre ectopique endothoracique est une maladie rare, il représente moins de 1% de l'ensemble des goitres endothoracique, son diagnostic positif est parfois difficile et repose sur les données de l'imagerie. Le traitement est chirurgical en raison des risques de compression et de dégénérescence maligne. Nous rapportant un cas particulier d'un goitre ectopique endothoracique prenant la forme d'une masse kystique du médiastin avec des signes de compression du nerf phrénique.

## Introduction

Le goitre ectopique endothoracique est une maladie rare, il représente moins de 1% de l'ensemble des goitres endothoracique. Il est défini par la présence d'une formation thyroïdienne de siège médiastinalavec sa propre vascularisation, sans connexion anatomique avec la glande cervicale et qui n'est pas la métastase d'un cancer thyroïdien. Nous rapportant un cas particulier d'un goitre ectopique endothoracique prenant la forme d'une masse kystique du médiastin avec des signes de compression du nerf phrénique.

## Patient et observation

**Figure 1:**
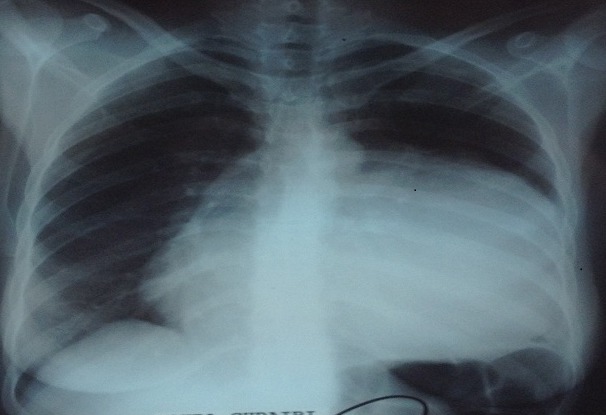
La radiographie du thorax montrant une opacité de tonalité hydrique occupant la moitié inférieure de l'hémi-champ pulmonaire gauche à bord externe en contacte avec la paroi, et à bord interne noyé dans le médiastin

**Figure 2:**
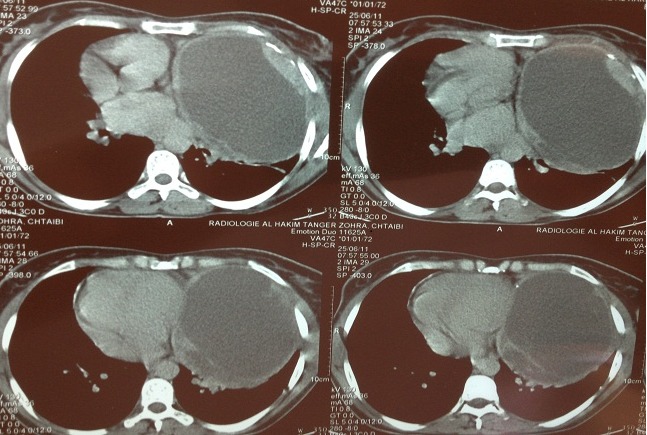
La tomodensitométrie thoracique avait montré la présence d'une masse médiastinale kystique à contenu épais, sans continuité avec la région cervicale

Patiente de 38 ans, sans antécédents pathologiques, qui présentait 3 mois avant son admission une douleur thoracique gauche, une dyspnée d'effort et une toux sèche. La radiographie du thorax montrait une opacité de tonalité hydrique occupant la moitié inférieure de l'hémi-champ pulmonaire gauche à bord externe en contacte avec la paroi, et à bord interne noyé dans le médiastin Figure 1. La tomodensitométrie thoracique avait montré la présence d'une masse médiastinale kystique à contenu épais comprimant le lingula, de 14 cm de diamètre antéropostérieur sur 11cm de largeur, avec une paroi épaisse refoulant le médiastin vers le coté droit Figure 2. Le bilan biologique était sans anomalies notamment la TSH qui était normale. Une résection chirurgicale était alors décidée vu le caractère compressif de la tumeur. La voie d'abord était une thoracotomie postérolatérale gauche. L'exploration chirurgicale avait trouvé une masse encapsulé du médiastin antérieur, sans prolongement cervical qui refoulait et comprimait le poumon, et dont la capsule adhérait intiment au nerf phrénique. Le geste chirurgicale était une résection complète de la masse et de sa capsule après une dissection minutieuse du nerf phrénique. Les suites opératoires étaient marquées par une parésie diaphragmatique qui avait régressée spontanément après trois mois. L'examen anatomopathologique avait confirmé le diagnostic d'un goitre multi-nodulaire bénin contenant une grande quantité d'un liquide noirâtre épais correspondant à du colloïde.

## Discussion

Les lésions kystiques du médiastin sont rares et représentent 12 à 30% des masses médiastinales, elles sont essentiellement d'origines congénitale [1]. Leurs étiologies sont multiples et sont principalement dominé par les kystes bronchogéniques qui représentent 50 à 60% de l'ensemble des kystes médiastinaux. On yretrouve également: les kystes para-œsophagiens, les kystes à paroi mésothéliale, les lymphangiomes kystiques, les kystes thymiques, les kystes parathyroïdien et les méningoceles [1]. Le goitre ectopique n'est pas une étiologie habituelle des masses kystiques médiastinales, on pense que cette présentation pourrait être expliquée par le contenu colloïde qui reste enfermé dans le tissu thyroïdien.

Le goitre endothoracique ectopique ou goitre primaire se développe à partir d′un tissu ectopique thyroïdien résultant d′une anomalie de sa migration lors de l′embryogénèse [2]. Il représente moins de 1% des goitres endothoraciques [2]. C'est plus fréquent chez la femme que chez l'homme, l’âge moyen de découverte est de45 ans [3]. Le développement se fait souvent dans le médiastin antérieur 75% à 94%, il se fait dans le médiastin postérieur dans 10% à 15% et il se fait plus du coté droit que du coté gauche.

L'euthyroïdie clinique et biologique est la règle chez la majorité des patients présentant un goitre ectopique mais des cas clinique d'hyperthyroïdie sont reporté dans la littérature [4]. Les goitres de petite taille peuvent rester longtemps asymptomatiques. Au moment du diagnostic plus de la moitié des goitres ectopiques sont symptomatiques [3]. Ces symptômes ne sont pas spécifiques est sont la conséquence d'une compression des organes de voisinage [5]. La douleur thoracique est retrouvée chez 71% des patients, une compression veineuse est retrouvée chez 57% et la dyspnée est retrouvée chez 43% des patients [3]. On peut avoir une dysphagie par compression œsophagienne ainsi qu'une compression nerveuse qui peut être responsable d'une parésie ou paralysie de la corde vocale, ou encore d'un syndrome de Claude Bernard-Horner [5], dans notre cas nous avons observé une parésie phrénique.

En effet chez notre patiente une ascension de la coupole diaphragmatique gauche était retrouvé en pré-opératoire, ce qui peut être expliquée par la compressiondu nerf phrénique.

La radiographie du thorax peut montrer une opacité hydrique du médiastin, la tomodensitométrie reste l'examen de choix en matière du diagnostic elle a une sensibilité de 100% pour Michel et all [6], en montrant une masse régulière, et hétérogène. L'imagerie par résonnance magnétique donne une étude morphologique fine du goitre et ses rapports. La supériorité de l'IRM sur la TDM a été suggérée par Janati et al dans le goitre médiastinal aberrant, et les goitres à composante vasculaire [5]. La scintigraphie peut aider au diagnostic on montrant une fixation à distance de la thyroïde cervical [2].

Une fois le diagnostic de goitre ectopique posé ou devant l'incertitude diagnostique la chirurgie doit être envisagée en raison du risque de compression des éléments thoraciques et du risque de malignité qui ne dépasse pas cependant celui des goitres cervicaux et qui est de l'ordre de 3-20% selon les auteurs [2, 4, 5]. La thyroïdectomie totale est le geste chirurgical indiquée dans les goitres endothoracique primaires. La cervicotomie est vouée à l’échec et est dangereuse en raison de la vascularisation d'origine thoracique [2]. La voie d'abord est fonction de la localisation du goitre.

## Conclusion

Le goitre ectopique intra-thoracique est rare, la présentation sous forme de masse kystique du médiastin est exceptionnelle, le diagnostic repose sur les données de l'imagerie et le traitement est chirurgical.
